# Cancer-Associated Mutations of the Adenosine A_2A_ Receptor Have Diverse Influences on Ligand Binding and Receptor Functions

**DOI:** 10.3390/molecules27154676

**Published:** 2022-07-22

**Authors:** Chenlin Feng, Xuesong Wang, Willem Jespers, Rongfang Liu, Sofía Denise Zamarbide Losada, Marina Gorostiola González, Gerard J. P. van Westen, Erik H. J. Danen, Laura H. Heitman

**Affiliations:** 1 Division of Drug Discovery & Safety, Leiden Academic Centre for Drug Research, Leiden University, 2333 CC Leiden, The Netherlands; c.l.feng@lacdr.leidenuniv.nl (C.F.); x.wang@lacdr.leidenuniv.nl (X.W.); w.jespers@lacdr.leidenuniv.nl (W.J.); r.liu@lacdr.leidenuniv.nl (R.L.); denise.zamarbide@hotmail.com (S.D.Z.L.); m.gorostiola.gonzalez@lacdr.leidenuniv.nl (M.G.G.); gerard@lacdr.leidenuniv.nl (G.J.P.v.W.); e.danen@lacdr.leidenuniv.nl (E.H.J.D.); 2 Oncode Institute, 2333 CC Leiden, The Netherlands

**Keywords:** adenosine A_2A_ receptor, cancer-associated mutation, GPCR, binding affinity, impedance-based cell-morphology assay, structure–activity relationship

## Abstract

The adenosine A_2A_ receptor (A_2A_AR) is a class A G-protein-coupled receptor (GPCR). It is an immune checkpoint in the tumor micro-environment and has become an emerging target for cancer treatment. In this study, we aimed to explore the effects of cancer-patient-derived A_2A_AR mutations on ligand binding and receptor functions. The wild-type A_2A_AR and 15 mutants identified by Genomic Data Commons (GDC) in human cancers were expressed in HEK293T cells. Firstly, we found that the binding affinity for agonist NECA was decreased in six mutants but increased for the V275A mutant. Mutations A165V and A265V decreased the binding affinity for antagonist ZM241385. Secondly, we found that the potency of NECA (EC_50_) in an impedance-based cell-morphology assay was mostly correlated with the binding affinity for the different mutants. Moreover, S132L and H278N were found to shift the A_2A_AR towards the inactive state. Importantly, we found that ZM241385 could not inhibit the activation of V275A and P285L stimulated by NECA. Taken together, the cancer-associated mutations of A_2A_AR modulated ligand binding and receptor functions. This study provides fundamental insights into the structure–activity relationship of the A_2A_AR and provides insights for A_2A_AR-related personalized treatment in cancer.

## 1. Introduction

The adenosine A_2A_ receptor (A_2A_AR), together with the other three subtypes of adenosine receptors (A_1_AR, A_2B_AR, and A_3_AR), belongs to the class A G-protein-coupled receptors (GPCRs) [1]. As common features of the GPCRs, the A_2A_AR has an extracellular N terminus, seven trans-membrane helices (TM1-TM7) connected by three intracellular loops (ICL1-ICL3) and three extracellular loops (ECL1-ECL3), and an intracellular C terminus [2]. When stimulated by its endogenous agonist, adenosine, or blocked by exogenous antagonists caffeine and theophylline, the A_2A_AR is involved in many physiological and pathological activities, including neurotransmission, blood-flow regulation, inflammation, and cancer [3].

The role of the A_2A_AR in cancer development has raised much interest in recent years and is highlighted by its immunosuppressive effects in the tumor micro-environment (TME). Adenosine is normally present at very low extracellular levels in healthy tissue [4], while in the TME, high levels of adenosine are present because more ATP is secreted due to cell damage and hypoxia and is further metabolized to AMP and adenosine [5]. The activation of the A_2A_AR on immune cells was found to suppress their anti-tumor responses, such as the inhibition of CD8+ T-cell activity [6], the inhibition of antigen presentation by dendritic cells [7], and the suppression of the cytotoxic function of NK cells [8]. Several in vivo studies have demonstrated the potential of small-molecule inhibitors as well as blocking antibodies targeting the A_2A_AR to treat cancer [9,10,11]. Thus, the A_2A_AR is an emerging immune checkpoint and a promising target for cancer treatment [12].

Thus far, several crystal structures of the A_2A_AR in complex with either an agonist or antagonist have been resolved [13,14,15]. However, most knowledge on the structure and function of the A_2A_AR, as well as drug discovery, is based on the wild-type receptor. Mutagenetic studies have been widely performed on the A_2A_AR and many other GPCRs, where residues in the ligand binding site were often replaced by alanine to identify key interactions of ligands with the wild-type receptor [16,17]. In previous research studies conducted by our group, we also used mutagenetic experiments to reveal the mechanism of antagonist dissociation from the A_2A_AR [18,19]. Nevertheless, we still lack knowledge on the ligand binding and functions of mutant A_2A_ARs, especially when these mutations occur in physiological conditions as natural variants or in pathological conditions as potential disease-driving factors. As for cancer, nearly 20% of human tumors contain mutations in genes encoding GPCRs [20], and many genes are statistically more frequently mutated relatively to the background mutation rate [21]. Therefore, further investigation on cancer-associated mutations would help to better understand the phenotypical and biological outcome of these mutations and could promote personalized drug discovery.

In this study, by exploring the sequencing data from the Genomic Data Commons data portal (GDC), we selected 15 A_2A_AR mutations found in cancer tissue and investigated their effects on ligand binding and receptor functions. Our results showed that six mutations decreased the binding affinity for agonist NECA compared with the wild-type A_2A_AR, while only one mutation increased the affinity. Two mutations were found to decrease the affinity for antagonist ZM241385. Besides, several mutations could alter the potency of NECA for receptor activation (EC50) or of ZM241385 for receptor inhibition (IC50), which was mostly correlated with changes in the binding affinity. This study is the first to systematically characterize the cancer-associated mutations of the A_2A_AR, and it pinpoints mutations that impact receptor activity and that may influence therapeutic strategies targeting the A_2A_AR.

## 2. Results

### 2.1. Selection of Cancer-Associated A_2A_AR Mutations

**Figure 1:**
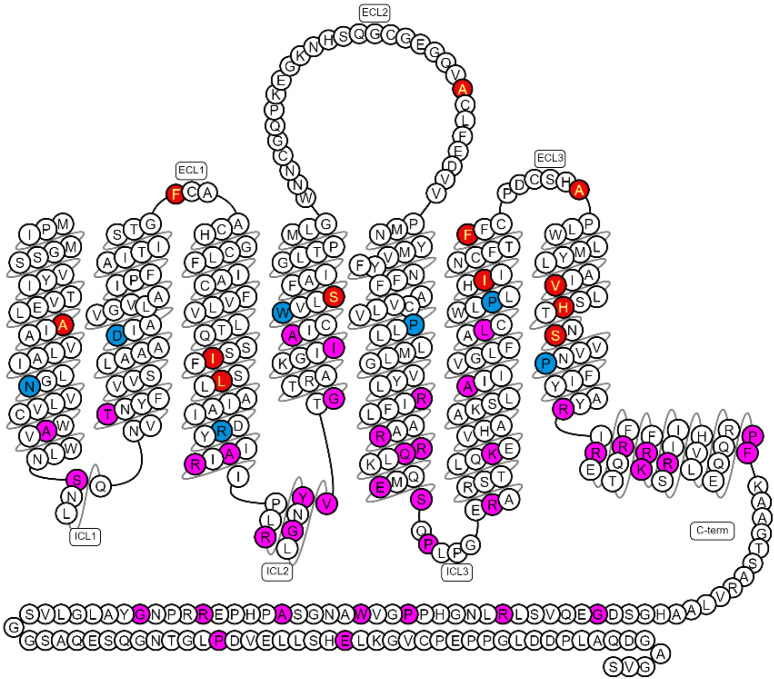
Snake plot showing the primary structure of wild-type human A_2A_AR. Blue: the most conserved *. 50 residues at each helix (Ballesteros–Weinstein numbering system). Red: residues with cancer-associated mutations in the upper part of the receptor (P^7.50^ is labeled in blue, where mutation P285L^7.50^ was identified). Magenta: residues with cancer-associated mutations in the lower part of the receptor. This figure was derived from GPCRdb.org [17].

From the Genomic Data Commons database (version 22.0; as collected by Bongers et al.) [22,23], a total of 58 A_2A_AR single-site missense mutations were identified in patients of different cancer types. As shown in Figure 1, these mutations were distributed all over the receptor. Among them, 15 mutations were located towards the extracellular region, as seen from the most conserved *. 50 residues (Ballesteros–Weinstein numbering system) [24], and the other mutations were in the lower part of the receptor. Since most A_2A_AR agonists and antagonists are extracellular ligands, it was inferred that mutations in the upper part were relatively close to the binding pocket and potentially involved in ligand binding or entry. Therefore, these 15 mutations were selected for further investigation. Of note, A265T^ECL3^ and S281L^7.46^ have also been identified as natural variants (The 1000 Genomes Project Consortium, as collected by Bongers et al.) [23,25], while the other mutations could be considered cancer specific (Table S1).

### 2.2. Validation of Expression and Radioligand Binding of Wild-Type and Mutant A_2A_ARs

**Figure 2:**
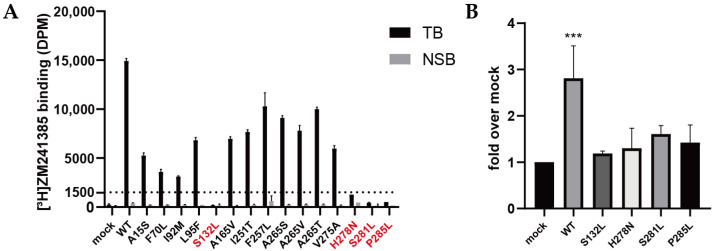
Radioligand binding and expression of wild-type and mutant A_2A_ARs. (**A**) Radioligand binding of 2.5 nM [^3^H]ZM241385 with 10 µg of membrane protein of HEK293T cells transiently transfected with wild-type or mutant A_2A_AR. Specific binding was defined as the difference between total binding (TB) and non-specific binding (NSB). NSB was determined with 100 μM NECA as displacer. Data are shown as mean ± SD of two independent experiments performed in duplicate. (**B**) Expression levels of transiently transfected WT and mutant A_2A_ARs on HEK293T-cell membrane, measured with an enzyme-linked immunosorbent assay. Mock values were measured with HEK293T cells transfected with a pcDNA3.1 empty vector as negative control. Data are shown as mean ± SD of two independent experiments performed in quintuplicate. (*** *p* < 0.001; ordinary one-way ANOVAs with Dunnett’s multiple comparisons tests; mock as control).

To validate the expression and radioligand-binding ability of A_2A_ARs, 2.5 nM [^3^H]ZM241385 was first applied for the wild-type and 15 mutant receptors of interest. The binding window was defined as the difference between total binding (TB) and non-specific binding (NSB), thus representing the specific binding of the radioligand to the (mutant) A_2A_ARs. As shown in Figure 2A, [^3^H]ZM241385 did not bind specifically to HEK293T membranes transfected with empty plasmids (mock), indicating that the endogenous expression of A_2A_AR was negligible compared with A_2A_AR-transfected samples (WT). All mutants displayed a lower specific binding of [^3^H]ZM241385 than the wild-type receptor, which suggested that they either had a lower expression level or [^3^H]ZM241385 bound to them with lower affinity. Of note, especially for S132L, H278N, S281L, and P285L mutants, a significantly decreased binding of [^3^H]ZM241385 was observed.

With an N-terminal FLAG-tagged construction of the receptors, an ELISA was performed to determine the expression level of the four mutants that displayed the lowest radioligand binding capacity, as shown in Figure 2B. The average expression level of the wild-type A_2A_AR was significantly higher (~2.8 fold) than that of mock HEK293T cells, while the expression levels of all four mutants were not significantly different from that of mock cells. These results indicated that the low expression of S132L, H278N, S281L, and P285L might have been the main reason why little binding of [^3^H]ZM241385 was observed. Consequently, the affinities for ZM241385 and NECA could not be determined for these mutants.

### 2.3. Quantification of Expression Levels (B_max_) and ZM241385 Binding Affinity (K_D_) for A_2A_ARs

**Figure 3:**
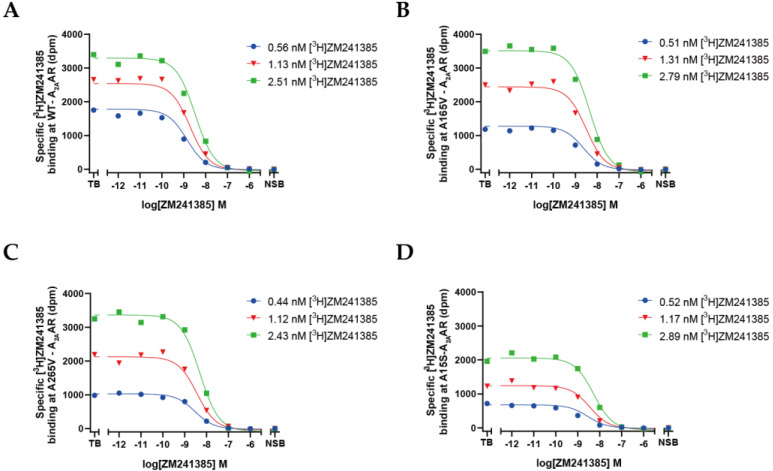
Homologous displacement of three concentrations of [^3^H]ZM241385 performed by increasing the concentrations of ZM241385 in WT-A_2A_AR (**A**), A165V-A_2A_AR (**B**), A265V-A_2A_AR (**C**), and A15S-A_2A_AR (**D**). Note that different concentrations of membranes were used depending on receptor expression levels, i.e., WT-A_2A_AR (1 μg), A165V-A_2A_AR (3 μg), A265V-A_2A_AR (1.5 μg), and A15S-A_2A_AR (3 μg). Three independent experiments were performed in duplicate, and representative curves from one experiment are shown above. Compared with WT-A_2A_AR, A165V and A265V displayed different affinity for ZM241385, and A15S displayed similar affinity but different B_max_. Graphs for other mutants can be found in Figure S1.

Radioligand-homologous-displacement experiments were performed to determine the affinity of ZM241385 and the receptor expression levels of wild-type and 11 mutant A_2A_ARs (Table 1). It was shown that ZM241385 bound to A165V (K_D_ = 2.2 nM; Figure 3B) and A265V (K_D_ = 3.1 nM; Figure 3C) with a lower affinity than that for the wild-type receptor (K_D_ = 0.98 nM; Figure 3A), while the pK_D_ values of the other nine mutants were not significantly different from that of the wild-type (Figure 3D and Figure S1), indicating that these mutations did not affect the binding affinity for ZM241385 (*p* > 0.05; ordinary one-way ANOVAs).

The expression levels of A_2A_ARs in transiently transfected HEK293T cells was presented as B_max_ (Table 1). Although all mutants were expressed at high levels after transient transfection, seven mutants showed significantly lower expression levels than that of the wild-type A_2A_AR (B_max_ = 37 pmol/mg), i.e., A15S (12 pmol/mg; Figure 3D), F70L (8 pmol/mg), I92M (9 pmol/mg), L95F (10 pmol/mg), A165V (17 pmol/mg), I251T (14 pmol/mg), and V275A (20 pmol/mg). The other four mutants (F275L, A265S, A265V, and A265T) showed expression levels similar to that of the wild-type receptor.

### 2.4. Quantification of NECA Binding Affinity (K_i_) for A_2A_ARs

**Figure 4:**
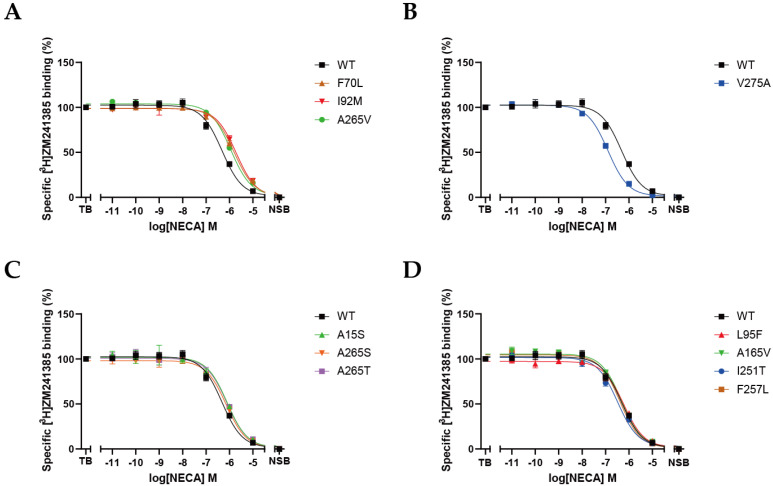
Displacement of [^3^H]ZM241385 performed by increasing the concentrations of NECA in wild-type and mutant A_2A_ARs. Compared with wild-type A_2A_AR, three mutants displayed lower affinity for NECA (**A**), V275A-A_2A_AR displayed higher affinity for NECA (**B**), three mutants displayed slightly different affinity for NECA (**C**), and four mutants displayed similar affinity for NECA (**D**). Data are shown as mean ± SEM of three independent experiments, each performed in duplicate.

The binding affinity of NECA for the wild-type and 11 mutant A_2A_ARs was determined with radioligand-heterologous-displacement experiments. Based on the results shown in Figure 4A and Table 1, F70L (K_i_ = 595 nM), I92M (K_i_ = 606 nM), and A265V (K_i_ = 632 nM) drastically decreased the binding affinity for NECA compared with the wild-type A_2A_AR (K_i_ = 134 nM). Besides, A15S, A265S, and A265T slightly but significantly decreased the binding affinity for NECA by approximately two-fold (Figure 4C). Interestingly, V275A (K_i_ = 42 nM; Figure 4B) was the only mutation that increased the affinity of NECA for the A_2A_AR.

### 2.5. Functional Effects of Cancer-Associated Mutations on A_2A_AR in a Label-Free Whole-Cell Assay

**Figure 5:**
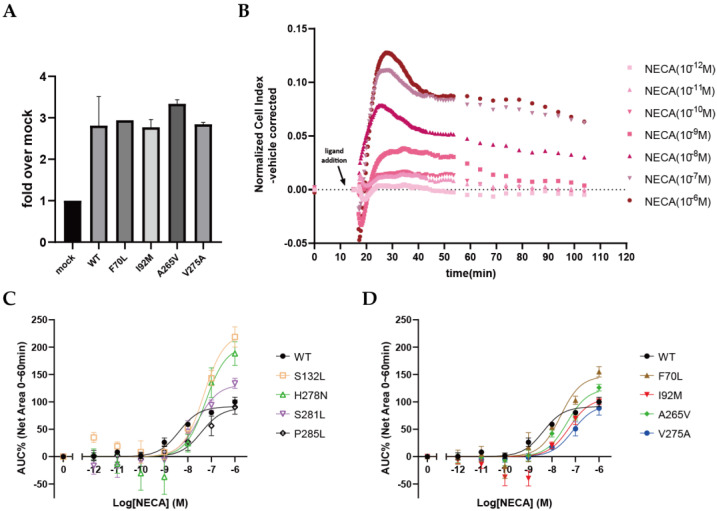
Functional characterization of wild-type and mutant A_2A_ARs via NECA stimulation of transiently transfected HEK293T cells, using a label-free impedance-based cell-morphology assay. (**A**) Expression levels of transiently transfected WT and mutant A_2A_ARs on HEK293T cells, measured with an enzyme-linked immunosorbent assay. Data are shown as mean ± SD of two independent experiments performed in quintuplicate. (**B**) Representative graph of vehicle-normalized cell index after stimulation with different concentrations of NECA of the wild-type A_2A_AR. Graphs of mutant A_2A_ARs are shown in Figure S2. (**C**,**D**) Concentration–response curves of NECA for wild-type and mutant A_2A_ARs derived from the area under the curve within 60 min after ligand addition. The response to vehicle was normalized to 0%, and the response to 1 μM NECA of the wild-type receptor was set to 100%. Data are shown as mean ± SEM of three independent experiments, each performed in duplicate.

To investigate the functional consequence of A_2A_AR mutations, HEK293T cells transiently transfected with either wild-type or mutant A_2A_ARs were used in the cell-morphology assay. Here, we used a label-free detection system (xCELLigence RTCA system), which was applied in our lab to quantify the activation level of the wild-type A_2A_AR in real-time, where we found that it was correlated to the more classic cAMP accumulation assay [26]. In total, eight mutants were selected for functional characterization, as these mutants either displayed over three-fold changes in the binding affinity for NECA (F70L, I92M, A265V, and V275A) or could not be assessed with binding experiments due to their low expression levels (S132L, H278N, S281L, and P285L; Figure 2). For the first set of four mutants, ELISA experiments were also performed, and the results indicated that they expressed mutant receptors at levels similar to that of the wild-type A_2A_AR (Figure 5A).

Next, the changes in cell morphology were monitored in real time after the stimulation of the cells with agonist NECA for the wild type and the selected eight mutants. In this label-free assay, cell-morphology changes affected the electronic readout of cell–sensor impedance, which was displayed as the cell index (CI). It is shown in Figure 5B that the CI of wild-type-A_2A_AR-transfected cells slightly decreased upon the addition of NECA and then sharply increased and reached a peak response within 10~15 min. Thereafter, the CI gradually decreased towards a plateau within 60 min and continued to slowly decrease towards baseline levels. The NECA-induced response was dose dependent (Figure 5B), with a potency of 8.4 ± 0.2 with respect to the wild-type A_2A_AR (Table 2).

Having established a wild-type A_2A_AR response for NECA in transiently transfected HEK293T cells, the above eight mutants were characterized following the same procedure. All eight mutants could be activated by NECA, resulting in a CI trace shape similar to that of the wild-type receptor (data not shown). The potency (EC_50_), intrinsic efficacy (E_max_), and relative efficacy (τ) of NECA for each A_2A_AR mutant were determined and are detailed in Table 2. NECA displayed a significantly decreased potency for almost all mutants when compared with the wild-type A_2A_AR (pEC_50_ = 8.4 ± 0.2), except for F70L (pEC_50_ = 7.6 ± 0.2) and S281L (pEC_50_ = 7.7 ± 0.3), for which the potencies were modestly decreased but not statistically different (*p* > 0.05; ordinary one-way ANOVAs). Notably, V275A, the only mutant with an increased binding affinity for NECA (Table 1), displayed the lowest potency (pEC_50_ = 7.1 ± 0.1). Moreover, S132L and H278N, which had much lower expression levels than the wild-type receptor (Figure 2B), both showed a significant increase in efficacy (E_max_% = 248 ± 30 for S132L, E_max_% = 223 ± 33 for H278N, and E_max_% = 100 ± 5 for WT). The intrinsic efficacies of the other mutants were not significantly different from that of the wild-type receptor. The relative efficacy was also calculated for each mutant receptor, where the affinity of NECA was taken into consideration. This resulted in a different ranking of NECA with respect to the different receptors compared with the intrinsic efficacy (Table 2). The wild-type A_2A_AR showed the highest relative efficacy (τ = 37 ± 13), followed by F70L (τ = 28 ± 11), A265V (τ = 21 ± 8), and I92M (τ = 10 ± 3), while V275A (τ = 1 ± 0) showed the lowest relative efficacy, indicating that this mutation caused a loss in coupling efficiency.

**Figure 6:**
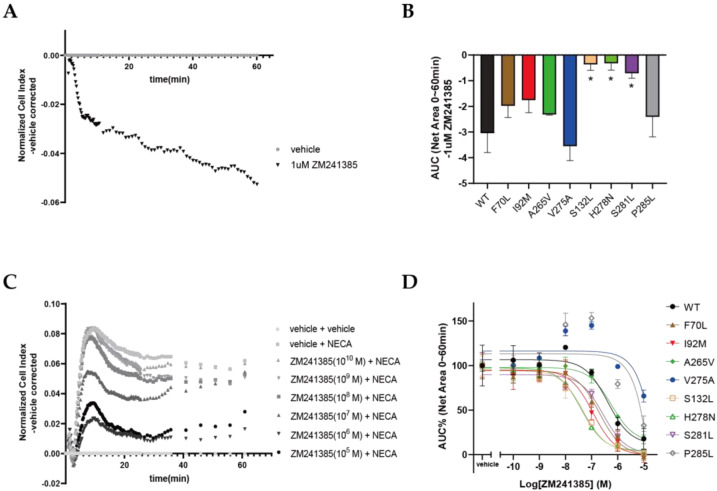
Functional characterization of wild-type and mutant A_2A_ARs with inverse agonist ZM241385 in transiently transfected HEK293T cells, using a label-free impedance-based cell-morphology assay. (**A**) Representative time trace of the normalized cell index of WT-A_2A_AR-expressing HEK293T cells stimulated with 1 μM ZM241385. (**B**) Area under the curve within 60 min after ZM241385 treatment was used to quantify its inverse agonism for A_2A_ARs. Data are shown as mean ± SEM of three independent experiments performed in duplicate. Significant difference from the WT-A_2A_AR is shown as * *p* < 0.05; ordinary one-way ANOVAs with Dunnett’s multiple comparisons tests. (**C**) Representative graph of normalized cell index of different concentrations of ZM241385 with respect to NECA-stimulated wild-type A_2A_AR. Vehicle was used for baseline correction. (**D**) Concentration–response curves of ZM241385 for wild-type and mutant A_2A_ARs derived from the area under the curve within 60 min after NECA addition. Response to vehicle was normalized to 0%, and response to EC_80_ of NECA after pretreatment with vehicle was normalized to 100%. Data are shown as mean ± SEM of three independent experiments, each performed in duplicate.

Besides agonist-dependent activation, the inhibition of A_2A_ARs by antagonist ZM241385 was also investigated. Since ZM241385 often shows inverse agonism on A_2A_AR, we first studied this pharmacological feature with an impedance-based assay. As shown in Figure 6A, after the addition of 1 µM ZM241385 to HEK293T cells transfected with wild-type A_2A_AR, the cell index first slightly increased; then, it sharply decreased within 5 min and further decreased slowly over time. Upon the normalization of the response to the vehicle, the area-under-curve value was thus negative, indicating that ZM241385 exhibited an opposite pharmacological effect towards agonist NECA (Figure 5B). The responses of wild-type and mutant A_2A_ARs to 1 μM ZM241385 were quantified and are displayed in Figure 6B and Table 3. Most mutants showed levels of inverse agonism similar to that of the wild-type A_2A_AR. However, mutants S132L, H278N, and S281L displayed a much lower level of inverse agonism than the wild-type A_2A_AR. Interestingly, NECA induced a higher level of activation in these mutants (Table 2), indicating that these mutations induced a conformation of the receptor that had less basal activity but was prone to higher levels of agonist-induced activation.

Lastly, we compared the inhibitory effects of ZM241385 on NECA-induced activation of the wild-type and different A_2A_AR mutant receptors. A dose-dependent inhibition of ZM241385 in wild-type A_2A_AR was observed, as shown in Figure 6C. Dose–response curves are depicted for all mutants in Figure 6D, and pIC_50_ values are shown in Table 3. Compared with wild-type A_2A_AR (pIC_50_ = 6.4 ± 0.3), ZM241385 displayed significantly higher potency with respect to S132L (pIC_50_ = 7.3 ± 0.1) and H278N (pIC_50_ = 7.4 ± 0.1). In addition, a small but significant increase in potency was found for F70L (pIC_50_ = 6.7 ± 0.2), I92M (pIC_50_ = 6.9 ± 0.1), and S281L (pIC_50_ = 6.6 ± 0.1), whereas the potency with respect to A265V (pIC_50_ = 6.3 ± 0.2) was similar to that with respect to the wild-type A_2A_AR. However, ZM241385 was not able to inhibit the NECA-induced activation of V275A and P285L, unless high concentrations were used. Of note, its affinity was not affected for V275A (and could not be determined for P285L; Table 1), indicating that its potency was negatively impacted by these mutations.

### 2.6. Mapping of Cancer-Associated Mutations in the Crystal Structures of A_2A_AR

**Figure 7:**
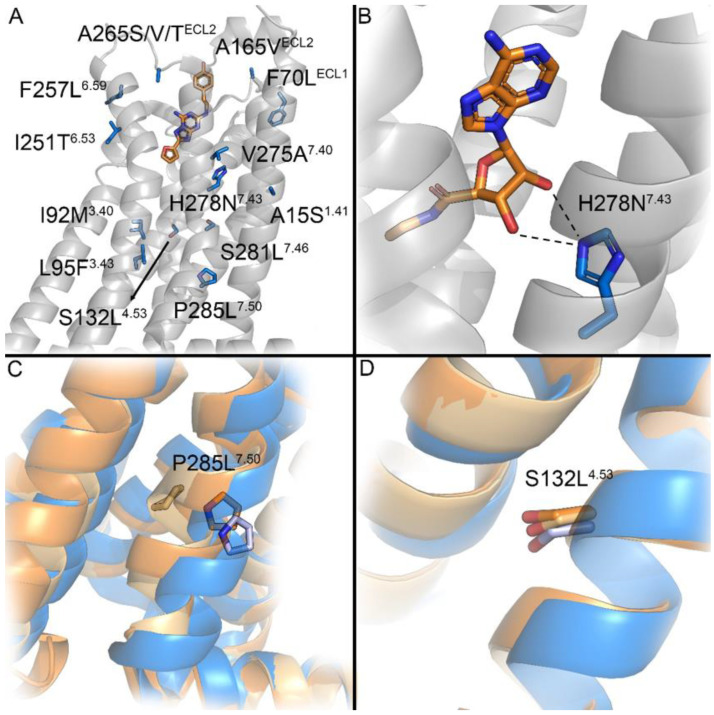
(**A**) Overview of all mutations investigated in this work, mapped on the inactive structure of the receptor (blue); antagonist ZM241385 is shown in orange. (**B**) Residue H278 was involved in agonist binding (NECA; orange), forming hydrogen bonds (dashed lines) with the ribose moiety. (**C**) P285 underwent extensive conformational rearrangement in the active-like structure (light orange). The active structure (orange) was closer to the inactive structure. (**D**) Residue S132 did not undergo extensive rearrangement when comparing inactive, active-like, and active structures.

To provide insight into the structure–activity relationship observed in the introduced mutants, we mapped each of the mutations on the available experimentally determined structures of the A_2A_AR, for which we used the inactive (PDB ID: 4EIY), active-like (PDB ID: 2YDV), and fully active (PDB ID: 5G53) structures. As shown in Figure 7A, the mutations were scattered around the binding pocket, though some clustering of residues could be observed, for instance, in TM7. We focused on mutations that abominated binding and were either in direct contact with the ligand in the binding pocket, i.e., H278N, or introduced large changes in the amino acid composition that might have influenced the activation of the receptor, e.g., S132L and P285L. H278 formed an extensive hydrogen-bond network with the ribose moiety of the agonist (Figure 7B). These hydrogen-bond patterns might have been impaired by the H278N mutation, which explains why the potency of NECA was significantly reduced for this mutant (Table 2). Residue P285 was located close to the center of TM7, which moved inward during the activation process of the A_2A_AR. As shown in Figure 7, TM7 was in a similar overall position both in the active-like state and fully active state, while the center of TM7 moved further inward in the active-like state, and the orientation of the helix in the fully active structure was closer to the inactive structure. It is well known that Pro residues introduce alpha-helical kinks, which might facilitate this movement. Therefore, in the P285L mutant, the substitution of Pro with Leu might have abrogated this conformational rearrangement of TM7, thus resulting in the decrease in the potency of both NECA and ZM241385 observed in the impedance-based assay (Table 2 and Table 3). Although mutation S132L was also shown to greatly decrease the potency of NECA, it did not undergo any structural rearrangements when comparing the different states of the crystal structure (Figure 7D) and was relatively far away from the binding site. Thus, the mechanism of the pharmacological effects of S132L remains to be revealed.

## 3. Discussion

Mutagenetic studies of the A_2A_AR have been performed since the 1990s and were later on complemented by computational modeling and crystallography [27]. From these studies, numerous A_2A_AR mutations are known to alter ligand binding and receptor activation [28,29]. In addition, it has been reported that impaired receptor expression is the most common defect caused by GPCR mutations, often combined with receptor instability and malfunction [30]. However, as A_2A_AR is emerging as a novel therapeutic target for cancer, little attention has been given to the cancer-associated mutations of the receptor and their potential pharmacological effects in the context of cancer biology and targeting. Therefore, in this study, 15 single-site mutations of A_2A_AR were retrieved from the Genomic Data Commons data portal [31]. These mutations were characterized for their expression levels and effects on ligand binding and receptor functions.

### 3.1. Some Cancer-Associated Mutations Cause a Conformational Change in A_2A_AR

Mutations S132L^4.53^ and H278N^7.43^ decreased the potency of NECA by over 10-fold, whereas E_max_ was increased by more than two-fold (Figure 5C). The decreased potency could have been caused by lower receptor expression, as higher agonist concentrations were required for exerting a certain level of biological activity [32]. In addition, residue His278^7.43^ was in direct contact with NECA via extensive hydrogen bonding (Figure 7B), and the mutation to Asn might have impaired this interaction. It was reported in a previous study that substitution of His278^7.43^ with either Ala, Lys, or Asn abolished the binding of agonists NECA and CGS21680 and antagonist XAC [29]. On the other hand, in this study, we observed that H278N^7.43^ could be activated by NECA in a dose-dependent manner, albeit at lower potencies than for the wild-type receptor. Hence, we infer that the binding of NECA with H278N^7.43^ occurs with a lower affinity but is not abolished. Different from this, S132L^4.53^ was located relatively far away from the binding site (Figure 7A); thus, the decreased potency may not have resulted from a change in ligand binding. Moreover, the effect of this mutation might have been caused by an overall destabilization of the receptor, as suggested by a much lower receptor expression. Further studies are needed to clarify the structural basis of altered receptor functions caused by S132L^4.53^, which is currently the mutation with the highest incidence among all cancer-associated A_2A_AR mutations in the GDC data portal. Interestingly, these two mutations not only increased E_max_ but also drastically decreased the level of inverse agonism via ZM241385 (Figure 6B, Table 3), showing less constitutive activity of the receptor, thus suggesting a more inactive conformation of the receptor. The constitutive activity of the A_2A_AR was previously reported, with A_2A_AR being found to display a native level of activation in the absence of any agonist and inverse agonists being preferentially bound to the inactive state to reduce the activity [33,34]. In addition, in comparison with the wild-type receptor, the potency of ZM241385 with respect to S132L^4.53^ and H278N^7.43^ was not impaired but increased (Figure 6, Table 3). Therefore, it seems that S132L^4.53^ and H278N^7.43^ shifted the conformational equilibrium of A_2A_AR towards the inactive state. However, this shift could not be observed in our static-structure analysis, but it could be modeled with more advanced methods, for example, free-energy simulation [35]. Of note, Gao et al. reported a sodium binding pocket in the A_2A_AR formed by His278^7.43^ and Glu13^1.39^ and showed that mutation H278Y^7.43^ reduced the negative allosteric effect of sodium ions on agonist binding [36]. Although a physiological concentration of NaCl was found to be sufficient to stabilize the inactive conformation of the A_2A_AR [37], it remains unknown whether the substitution of His278^7.43^ with Asn instead of Tyr would augment the allosteric effect of sodium ions to achieve this effect.

S281^7.46^ was also investigated in other mutagenetic studies; for instance, Kim et al. found that the substitution of Ser with Ala abolished the binding of agonist GGS21680 and antagonist XAC, while the substitution with Thr increased the affinity for NECA, GGS21680, and XAC [28]. Besides, Jiang et al. reported that the substitution of Ser281^4.53^ with Asn increased the affinity for NECA and CGS21680 but decreased the affinity for ZM241385 [38]. These studies suggested that the hydrophilic side chain of S281^7.46^ is beneficial to ligand recognition. Although we could not determine the binding affinity values for this mutant due to its low expression level, it is inferred that hydrophobic mutation S281L^7.46^ could cause a decrease in the affinity for NECA, which would be consistent with the slightly decreased potency observed in the cell-morphology assay. Moreover, we observed that mutation S281L^7.46^ decreased the inverse agonism induced by ZM241385, indicating a more inactive conformation of the receptor. This might be explained by the fact that S281^7.46^ is positioned in the allosteric sodium binding site of the A_2A_AR (D52^2.50^, S91^3.39^, T88^3.36^, W246^6.48^, N280^7.45^, and S281^7.46^) [15,37].

P285^7.50^ is located in the highly conserved NPxxY motif that is known to be involved in GPCR activation [2,39]. Massink et al. found that mutation N284A^7.49^ in the A_2A_AR completely abolished receptor activation [40]. However, in our study, mutation P285L^7.50^ caused a 10-fold decrease in the potency of NECA but similar efficacy with respect to the wild-type A_2A_AR, indicating that the activation of the receptor was also impaired albeit not abolished. Based on structural mapping (Figure 7C), P285^7.50^ showed a drastic conformational rearrangement during the receptor activation process, which might be impeded if Pro were substituted with Leu. Interestingly, ZM241385 could only inhibit the activation of the P285L^7.50^ mutant at concentrations above 1 μM (Figure 6D). Although the affinity of ligands for P285L^7.50^ could not be determined, it was reported that mutation P285C^7.50^ in A_1_AR slightly increased the affinity for its antagonist DPCPX [41]. Considering the significantly decreased potency of antagonist ZM241385, we speculate that P285L^7.50^ may increase the affinity for NECA; thus, the competitive binding between the agonist and the antagonist would result in altered receptor inhibition. However, P285^7.50^ was located about 15 Å below the orthosteric binding site of NECA in the sampled conformational states. As this large distance could not allow this residue to interact with the bound ligand independently, further studies are needed to interpret its pharmacological effects. At last, compared with wild-type A_2A_AR, P285L^7.50^ displayed similar E_max_ of NECA and similar inverse agonism of ZM241385, so the conformational equilibrium did not seem to be influenced.

### 3.2. Some Cancer-Associated Mutations Affect Ligand Binding and Functioning of A_2A_AR

Looking into the structural features of these mutations and their effects on ligand binding, we found that all mutations located in the extracellular loops played a role. For example, F70L^ECL1^ decreased the affinity for NECA by around four-fold, and A165V^ECL2^ slightly decreased the affinity for ZM241385 and NECA, while A265V^ECL3^ drastically decreased the affinity for both ligands. Besides, although these three mutations all preserved the hydrophobic side chain, they still showed a considerable impact on the affinity, possibly due to an increase in the steric hindrance during ligand entry. Note that three different mutations were found in Ala265^ECL3^, i.e., A265S, A265V and A265T, which was located next to His264^ECL3^. His264^ECL3^ is part of the orthosteric binding pocket of A_2A_AR, and was found to have an aromatic interaction with the 4-hydroxyphenyl ring of ZM241385 [42]. Guo et al. also reported a hydrogen-bond network formed by His264^ECL3^, Glu169^ECL2^, and Thr256^6.58^ with ZM241385, and the disruption of this network by the mutation of either of these residues decreased the binding affinity for ZM241385 due to a faster dissociation rate of the antagonist from the receptor [18]. These studies are in line with our findings that the introduction of a bulkier hydrophobic side chain into Ala265 by substitution with Valine might cause stronger steric hindrance to His264 and thus a loss in the affinity for both the agonist and antagonist.

Of further interest is the mutations’ effects on receptor functions. For F70L^ECL1^, I92M^3.40^, and A265V^ECL3^, a decreased potency with respect to NECA was observed (Figure 5D, Table 2), which was correlated to a decreased binding affinity for this agonist (Figure 4A, Table 1). To be noted, residue I92^3.40^ constitutes the highly conserved PIF motif, which is an important micro-switch in class-A-GPCR activation [43]. Consistently, the decrease in the potency of I92M^3.40^ was larger than those of F70L^ECL1^ and A265V^ECL3^, resulting in a lower relative efficacy. The relative efficacy, τ, reflects the ability of a specific agonist to activate the receptor in relation to its receptor occupancy level (so more system independent) [44]. When τ is large, resulting from an agonist with a much higher potency than its affinity and with 100% E_max_, this indicates full agonism. Vice versa, small τ or lower E_max_ indicates partial agonism. As such, although the apparent maximal response to NECA of V275A^7.40^ was similar to that of the wild-type A_2A_AR (Figure 5D, Table 2), when calculating its relative efficacy, NECA seemed to act as a partial agonist. V275A^7.40^ was the only mutation that increased the affinity for NECA but decreased its potency. In other words, the activation of this mutant receptor by NECA was dependent on a larger proportion of receptor occupancy; thus, this indicated a loss in the coupling efficiency of the intracellular signaling pathways. Similar effects were described for VEGFR and β adrenergic receptors, whereby agonists may show “pluridimensional efficacy” depending on the level of receptor expression, the cell background, and the observed signaling pathway [45,46]. This and our data substantiate the importance of calculating the relative efficacies when comparing agonists and receptor variants, especially when using (transient) heterologous expression systems. In addition, ZM241385 could only exert inhibitory effects on V275A^7.40^ at high concentrations (Figure 6D, Table 3), resulting from the increased affinity for NECA and unaffected affinity for ZM241385 (Figure 4B, Table 1), which followed the same pattern observed in P285L^7.50^. Importantly, the lost potency of prototypic drug ZM241385 with respect to these two mutations indicates that they could be problematic in cancer treatment and deserve follow-up studies.

### 3.3. Potential Effects of A_2A_AR Mutations on Cancer Development and Treatment

Besides acting as an important checkpoint in immune cells, the antagonism of A_2A_AR signaling in tumor cells was also found to inhibit their growth [47,48]. This indicates that mutations influencing the ligand binding affinity or receptor activation level could change cancer cell growth or sensitivity to A_2A_AR-targeted treatment. For example, S132L^4.53^ and H278N^7.43^, which were observed to stabilize the inactive state of A_2A_AR, could inhibit the cell growth brought forth by the constitutive activation. However, considering the high concentrations of adenosine present in the TME [12] and the increased efficacy of agonist NECA with respect to these two mutants, cell growth could eventually be promoted, which means that these mutations could be oncogenic drivers. To be noted, the activation of these two mutants could still be inhibited by ZM241385 even with a higher potency (Table 3) and could render the cells more sensitive to the antagonism of the A_2A_AR. On the contrary, cells bearing the V275A^7.40^ or P285L^7.50^ mutation were likely to be resistant to prototype drug ZM241385 (Table 3); thus, a higher dose or more potent antagonists are needed when targeting these mutant receptors.

### 3.4. Conclusions

In this study, we characterized 15 cancer-associated mutations of the human adenosine A_2A_ receptor to study their effects on ligand binding and receptor functions in HEK293T cells. Several mutations were found to affect the binding affinity for an agonist or antagonist, the receptor expression level, and the constitutive activity of the receptor, as well as receptor activation and inhibition by a reference agonist and antagonist, respectively. This study provides novel fundamental insights into the structure–activity relationship of the adenosine A_2A_ receptor. Based on these findings, further studies in cancer-cell models are required to reveal the role of these A_2A_AR mutations in cancer progression. Moreover, identifying antagonists that affect wild-type as well as mutant receptors may lead to optimized therapeutic strategies targeting the A_2A_AR.

## 4. Materials and Methods

### 4.1. Chemicals and Reagents

[^3^H]ZM241385 (specific activity of 50 Ci/mmol) was purchased from American Radiolabeled Chemicals Inc. (St. Louis, MO, USA). Unlabeled ZM241385, 5′-Nethylcarboxamidoadenosine (NECA), and adenosine deaminase (ADA) were purchased from Merck Life Science N.V. (Amsterdam, The Netherlands). The BCA protein assay reagent was obtained from Fisher Scientific (Landsmeer, The Netherlands). Quick Change II Site-Directed Mutagenesis Kit was purchased from Agilent Technologies Netherlands B.V. (Amstelveen, The Netherlands). All other chemicals were of analytical grade and obtained from standard commercial sources.

### 4.2. Site-Directed Mutagenesis

Plasmid DNA of A_2A_AR mutants were constructed using polymerase chain reaction (PCR) based on pcDNA3.1(-)-A_2A_AR-wt with N-terminal FLAG tag and C-terminal His tag as template, using Quick Change II Site-Directed Mutagenesis Kit. Mutagenesis primers for PCR cloning were designed using the online Quickchange primer design tool (Agilent Technologies) and synthesized by Integrated DNA Technologies (Coralville, CA, USA). All DNA sequences were verified using Sanger sequencing at Leiden Genome Technology Center (Leiden, The Netherlands).

### 4.3. Cell Culture and Transfection

Human embryonic kidney 293T (HEK293T) cells were grown in Dulbecco’s modified Eagle medium (high glucose) supplemented with 10% fetal calf serum, 200 μg/mL penicillin and 200 μg/mL streptomycin, at 37 °C and 5% CO_2_. Cells were subcultured at a ratio of 1:15 twice a week on 10 cm ø plates. Before transfection, cells were subcultured at a ratio of 1:8, and after 24 h of proliferation, they could reach ~50% confluency. Cells were transfected with wild-type or mutant pcDNA3.1(-)-A_2A_AR plasmid DNA (1 μg/plate) using the calcium phosphate precipitation method [49]. In short, 1 μg of plasmid DNA was dissolved in 365 μL of water and then mixed with 135 μL of 1M CaCl_2_ solution. The mixture was added dropwise to a HBSS buffer under aeration to form a fine precipitate, which was applied at 1 mL/plate to HEK293T cells. The HBSS buffer contained 280 mM NaCl, 10 mM KCl, 1.5 mM Na_2_HPO_4_ and 50 mM HEPES, pH 7.05.

### 4.4. Enzyme-Linked Immunosorbent Assay (ELISA)

To determine the expression level of wild-type or mutant A_2A_ARs on HEK293T-cell membranes, 24 h after transfection, cells were detached with PBS/EDTA and resuspended in culture medium. Cells were then seeded in poly-D-lysine coated 96-well plates at a density of 1 × 10^6^ cells/well in quintuplicate. After 24 h of incubation at 37 °C and 5% CO_2_, the medium was removed, and cells were washed with PBS and subsequently fixed with 100 μL/well 4% formaldehyde for 10 min. Cells were washed with TBS (20 mM Tris-HCl, 150 mM NaCl (pH 7.5)) twice before 100 μL/well blocking buffer (2% *w*/*v* bovine serum albumin in TBST) was added, and they were incubated for 30 min at room temperature. After that, the blocking buffer was removed, and cells were incubated with 100 μL/well primary antibody (mouse anti-FLAG monoclonal antibody; Sigma F3165, Merck Life Science NV, Amsterdam, Netherlands; 1:4000) for 2 h at room temperature while being shaken at 300 rpm. Next, the primary antibody was removed, and cells were washed with TBST (TBS with 0.1% Tween-20) three times before the addition of 100 μL/well secondary antibody (goat anti-mouse IgG HRP conjugated; Jackson ImmunoResearch Laboratories 115-035-003; 1:10,000) and were incubated for 1 h at room temperature while being shaken. After the removal of the secondary antibody, cells were washed three times with TBS. Next, cells were treated with 100 μL/well 3, 3′, 5, 5′-tetramethylbenzidine (TMB; Sigma T0440) for 5 min in the dark; then, the reaction was stopped with the addition of 100 μL/well 1 M H_3_PO_4_ solution. Immediately after that, the absorbance was measured at 450 nm using an EnVision™ (Menlo Park, CA, USA) microplate reader. HEK293T cells transfected with vector pcDNA3.1 plasmid were used as a control (mock), the absorbance value of which was normalized to 1 for data analyses.

### 4.5. Membrane Preparation and Determination of Specific [^3^H]ZM241385 Binding

The preparation of cell membranes over-expressing A_2A_ARs for radioligand-binding assays was performed as previously reported [18]. Briefly, HEK293T transiently expressing the wild-type or mutant A_2A_AR were detached by scraping into PBS. Cell pellets were collected by centrifugation at 1000 rpm for 10 min to remove PBS and re-suspended in ice-cold Tris-HCl buffer (50 mM; pH = 7.4) prior to homogenization. The homogenized suspensions were centrifuged at 100,000× *g* for 20 min at 4 °C and re-suspended in Tris-HCl buffer to repeat the homogenization–centrifugation cycle again. At last, membranes from ten 10 cm ø plates were re-suspended in 1 mL of assay buffer (50 mM Tris-HCl, 5 mM MgCl_2_, 0.1% CHAPS) as used in radioligand-binding assays, homogenized, and treated with adenosine deaminase (0.8 IU/mL) to degrade endogenous adenosine. Membranes were stored in 100–250 μL aliquots at −80 °C. Membrane protein concentrations were determined using the BCA method [50].

To determine the binding capacity of [^3^H]ZM241385 with A_2A_ARs, 25 μL of membrane (10 μg protein/well) was mixed in a total volume of 100 μL, with the presence of 2.5 nM [^3^H]ZM241385, with 100 μM NECA to determine non-specific binding (NSB) or without NECA to determine total binding (TB). The mixtures were incubated at 4 °C for 2 h while being shaken at 200 rpm. Incubation was terminated with rapid vacuum filtration to separate the bound and free radioligands with a 96-well GF/C filter using Filtermate-harvester (PerkinElmer, Groningen, The Netherlands). Filters were washed ten times with ice-cold wash buffer (50 mM Tris-HCl, 5 mM MgCl_2_) before drying at 55 °C in an oven for 30 min. To measure the membrane-bound radioactivity, 25ul MicroScint™-20 cocktail was added to each well, and the filter was measured with MicroBeta^2^ Microplate Counter (PerkinElmer, Groningen, The Netherlands).

### 4.6. Radioligand-Homologous- and -Heterologous-Displacement Assays

For the homologous-displacement assay, increasing concentrations (from 10^−^^12^ M to 10^−^^6^ M) of unlabeled ZM241385 were used to displace the binding of three concentrations of [^3^H]ZM241385, i.e., 0.5 nM, 1 nM, and 2.5 nM, which were distributed around the estimated K_D_ of ZM241385 with A_2A_AR. For the heterologous-displacement assay, increasing concentrations (from 10^−^^11^ M to 10^−^^5^) of NECA were used to displace the binding of 2.5 nM [^3^H]ZM241385. Based on the pre-determined [^3^H]ZM241385 binding capacity for the different mutants, 25 μL of membrane aliquots containing 1~20 μg protein was used to adjust the total binding with 2.5 nM [^3^H]ZM241385 to approximately 2000 dpm and non-specific binding less than 10% of total binding. Membranes were incubated with the radioligand and the compound of interest in a total volume of 100 μL of assay buffer (50 mM Tris-HCl, 5 mM MgCl_2_, 0.1% CHAPS), as described above.

### 4.7. Label-Free Whole-Cell Assay (xCELLigence RTCA System)

The functional characterization of transiently transfected wild-type and mutant A_2A_ARs was performed on HEK293T cells with the xCELLigence RTCA system, as previously described [51]. Briefly, an arrayed microelectrode was embedded at the bottom of each well of a 96-well E-plate (Roche Applied Science, Mannheim, Germany). During cell spreading and proliferation, the cell-morphology changes affected the electronic readout of cell–sensor impedance (Z), which was monitored in real time by the xCELLigence RTCA system and displayed as the cell index (CI). If the cells were stimulated by a ligand, the changes in the CI reflected the overall cellular response upon the activation of GPCR-mediated signaling.

To study the stimulation of A_2A_ARs with NECA, HEK293T cells were transfected with the wild-type or mutant A_2A_AR following the methods described above. A total of 24 h after transfection, cells were detached with PBS/EDTA and suspended in culture medium. The cell suspension was centrifuged at 1000 rpm for 5 min to remove the supernatant; then, cell pellets were re-suspended in culture medium to adjust the concentration to 1 × 10^6^ cells/mL. First, 50 μL of culture medium was added to each well of a 96-well E-plate to measure the background (Z_0_). Next, 40 μL of cell suspension containing 40,000 cells was added to each well, and the E-plate was left at room temperature for 30 min before being placed on the recording device station in the incubator at 37 °C and 5% CO_2_. The cells were cultured for 17~20 h until the end of the log phase, during which the CI was continuously measured every 15 min. After that, 5 μL adenosine deaminase solution (ADA; 2.5 IU/mL) was added to each well and incubated for 1.5 h to remove the adenosine present in the culture medium. Subsequently, cells were stimulated with 5 μL of NECA (final concentration ranges from 10^−^^12^ M to 10^−^^6^ M) or vehicle control (final concentration of 0.1% DMSO). The changes in the CI after agonist addition were measured every 15 s within the first 30 min, followed by every 5 min up to 120 min. For data analyses, the CI of each group was normalized by subtracting the baseline (vehicle control) to correct for any non-specific signals. Dose–response curves were generated from the area under the curve (AUC) within the first 60 min after agonist addition, and parameters including EC_50_, EC_80_, and E_max_ were calculated to describe the potency and efficacy of NECA stimulation of wild-type or mutant A_2A_ARs.

To characterize the pharmacological effects of ZM241385 on wild-type or mutant A_2A_ARs, the experiments were performed similarly to those described above. Cells were then treated with 5 μL of ZM241385 (final concentration ranges from 10^−^^10^ M to 10^−^^5^ M) or vehicle control (final concentration of 0.05% DMSO), and the changes in the CI were measured every 15 s within the first 10 min and every 1 min up to 90 min. After that, 5 μL of NECA (final concentration equals to the EC_80_ of NECA for each A_2A_AR variant) or vehicle control (final concentration of 0.05% DMSO) was added to each well. For data analyses, the CI of each group was normalized by subtracting the baseline (vehicle control) to correct for any non-specific signals. The AUC within 60 min after compound addition was used to describe the initial response of ZM241385 itself and the inhibitory effects of ZM241385 upon NECA stimulation.

### 4.8. Data Analysis

All experimental data were analyzed using GraphPad Prism 9.0 (GraphPad Software Inc., San Diego, CA, USA), and the values obtained were means of two or three independent experiments. SD values were calculated for the results of two independent experiments (radioligand binding window check and ELISA), and SEM values were calculated for three independent experiments.

K_D_ values of [^3^H]ZM241385 obtained from homologous-displacement assays were calculated using nonlinear-regression curve fitting (Binding-Competitive-One site-Homologous), where 3 concentrations of [^3^H]ZM241385 were input and K_D_ was obtained with global fitting. *B_max_* values were obtained from homologous-displacement assays in “*dpm*”, and converted to *pmol*/*mg* using the following equations:

$$B_{max}\;\left(Ci\right)=\frac{Bmax\;\left(dpm\right)}{2.22\;\times \;10^{-12}}$$

$$B_{max}\;\left(mmol\right)=B_{max}\;\left(Ci\right)/Specific\;activity\;\left(Ci/mmol\right)$$

$$Specific\;activity=Specific\;activity\;at\;t_{0}\times \;0.5^{\frac{time\;lapse\;since\;prodection\;date}{half\;life\;of\;radio\;label}}$$

$$B_{max}\;\left(pmol/mg\right)=\frac{B_{max}\;\left(mmol\right)\;\times \;10^{9}}{Amout\;of\;membrane\;protein\;per\;well\;\left(mg\right)}$$

*IC*_50_ values obtained from heterologous-displacement assays were calculated by nonlinear-regression curve fitting using a one-site competitive binding model, where *K_D_* values were taken from the homologous-displacement assays for each receptor variant, and *K_i_* were converted from *IC*_50_ following the Cheng–Prusoff equation [52]:

$$K_{i}=\frac{IC_{50}}{1+\frac{\left[Radioligand\right]}{K_{D}}}$$

EC_50_ and *E_max_* values in the cell-morphology assay were obtained by plotting the normalized CI traces using RTCA Software 2.0 (Agilent Technologies Netherlands BV, Amstelveen, Netherlands). Dose–response curves were generated by calculating the area under curve over the first 60 min after compound addition and were analyzed using nonlinear-regression fitting (three-parameter model) to determine EC_50_ and *E_max_*. The relative efficacies (*τ*) of the agonist on each receptor variant were obtained by fitting the data to the operational model by Black and Leff [53], which correlates the biological effect, *E,* with agonist concentration [*A*]:

$$E=\frac{E_{max}\cdot \tau \cdot \left[A\right]}{K_{D}+\left(\tau +1\right)\cdot \left[A\right]}$$

### 4.9. Structural Mapping

Figures were created based on the experimentally determined structures for the A_2A_AR crystal structures, with PDB codes 4EIY [15] for the inactive structure, 2YDV [14] for the active-like structure and 5G53 [54] for the fully active structure. Figures were generated using PyMOL Molecular Graphics System version 2.0 (Schrödinger, LLC., New York, NY, USA).

## Figures and Tables

**Table 1:** Affinity values of ligands for wild-type (WT) and mutant A_2A_ARs and their expression levels (B_max_).

Mutant	ZM241385 pK_D_ ^a^ (K_D_ (nM))	NECA pK_i_^b^ (K_i_ (nM))	B_max_^a^ (pmol/mg)
WT	9.0 ± 0.1 (0.98)	6.9 ± 0.0 (134)	37 ± 5
A15S^1.41^	8.9 ± 0.1 (1.5)	6.6 ± 0.1 ** (277)	12 ± 2 ****
F70L^ECL1^	8.8 ± 0.1 (1.7)	6.2 ± 0.1 **** (595)	8 ± 1 ****
I92M^3.40^	8.9 ± 0.1 (1.3)	6.2 ± 0.1 **** (606)	9 ± 1 ****
L95F^3.43^	9.1 ± 0.0 (0.83)	6.9 ± 0.1 (144)	10 ± 1 ****
A165V^ECL2^	8.7 ± 0.1 ** (2.2)	6.7 ± 0.0 (210)	17 ± 1 ***
I251T^6.53^	8.8 ± 0.1 (1.9)	6.9 ± 0.1 (134)	14 ± 1 ***
F257L^6.59^	8.8 ± 0.0 (1.8)	6.8 ± 0.0 (151)	27 ± 1
A265S^ECL3^	8.9 ± 0.0 (1.3)	6.6 ± 0.1 * (246)	27 ± 6
A265V^ECL3^	8.5 ± 0.1 *** (3.1)	6.2 ± 0.0 **** (632)	39 ± 1
A265T^ECL3^	8.9 ± 0.0 (1.2)	6.6 ± 0.0 ** (266)	37 ± 6
V275A^7.40^	8.9 ± 0.1 (1.4)	7.4 ± 0.0 **** (42)	20 ± 2 **

^a^ pK_D_ and B_max_ were determined with a homologous displacement assay, where three concentrations of [^3^H]ZM241385 were displaced by increasing the concentrations of ZM241385. ^b^ pK_i_ values were determined with a heterologous displacement assay, where [^3^H]ZM241385 was displaced by increasing the concentrations of NECA. Values for S132L^4.53^, H278N^7.43^, S281L^7.46^, and P285L^7.50^ were not determined because little binding of [^3^H]ZM241385 was observed. Data are represented as mean ± SEM of three independent experiments performed in duplicate. (Significant difference from the wild type is shown as * *p* < 0.05, ** *p* < 0.01, *** *p* < 0.001, **** *p* < 0.0001; ordinary one-way ANOVAs with Dunnett’s multiple comparisons tests.)

**Table 2:** Potency and efficacy of NECA stimulation of the wild-type (WT) and mutant A_2A_ARs derived from cell-morphology-assay results.

Mutant	Potency pEC_50_ ^a^	Efficacy E_max_% (AUC-1 μM) ^a^	Relative Efficacy τ ^b^
WT	8.4 ± 0.2	100 ± 5 (5.7)	37 ± 13
F70L^ECL1^	7.6 ± 0.2	164 ± 19 (8.9)	28 ± 11
I92M^3.40^	7.2 ± 0.2 **	119 ± 4 (5.8)	10 ± 3
A265V^ECL3^	7.4 ± 0.2 *	135 ± 2 (7.2)	21 ± 8
V275A^7.40^	7.1 ± 0.1 **	106 ± 19 (5.1)	1 ± 0 *
S132L^4.53^	7.3 ± 0.0 *	248 ± 30 *** (12.6)	n.a.
H278N^7.43^	7.3 ± 0.2 **	223 ± 33 ** (10.8)	n.a.
S281L^7.46^	7.7 ± 0.3	146 ± 25 (7.7)	n.a.
P285L^7.50^	7.4 ± 0.5 *	101 ± 8 (5.2)	n.a.

^a^ Log potency (pEC_50_) and efficacy (E_max_%) of NECA were calculated from concentration–response curves derived from the area under the curve of CI changes within 60 min after stimulation. The AUC of CI changes within 60 min after 1 μM NECA addition is shown in brackets. ^b^ Relative efficacy was analyzed with the operational model by Black and Leff (1983) using global fitting. n.a. = not applicable; relative efficacy could not be determined as no affinity was obtained for NECA in these mutants. Data are represented as mean ± SEM of three independent experiments performed in duplicate. (Significant difference from the wild type is shown as * *p* < 0.05, ** *p* < 0.01, *** *p* < 0.001; ordinary one-way ANOVAs with Dunnett’s multiple comparisons tests.)

**Table 3:** Quantification of the inverse agonism of ZM241385 on A_2A_ARs and the inhibitory potency of ZM241385 on NECA stimulation.

Mutant	Inverse Agonism of	Inhibition
	ZM241385-1 μM ^a^	pIC_50_ ^b^
WT	−3.0 ± 0.8	6.4 ± 0.3
F70L^ECL1^	−2.0 ± 0.5	6.7 ± 0.2
I92M^3.40^	−1.8 ± 0.5	6.9 ± 0.1
A265V^ECL3^	−2.3 ± 0.0	6.3 ± 0.2
V275A^7.40^	−3.6 ± 0.6	n.d.
S132L^4.53^	−0.4 ± 0.2 **	7.3 ± 0.1 **
H278N^7.43^	−0.3 ± 0.3 **	7.4 ± 0.1 **
S281L^7.46^	−0.7 ± 0.2 *	6.6 ± 0.1
P285L^7.50^	−2.4 ± 0.8	n.d.

^a^ In the whole-cell-based cell-morphology assay, inverse agonism was calculated from the AUC of CI changes within 60 min after ZM241385 addition. ^b^ Cells were pretreated with increasing concentrations of ZM241385 before stimulating with EC_80_ of NECA. The inhibitory potency of ZM241385 (pIC_50_) was calculated from concentration–response curves derived from the area under the curve of CI changes within 60 min after NECA addition. n.d. = not determined, as no sigmoidal inhibition curve could be observed for the mutants. Data are represented as mean ± SEM of three independent experiments performed in duplicate. (Significant difference from the wild type is shown as * *p* < 0.05, ** *p* < 0.01; ordinary one-way ANOVAs with Dunnett’s multiple comparisons tests.)

## Data Availability

The data that support the finding of this study are available on request from the corresponding author, upon reasonable request.

## Supplementary Materials

The following supporting information can be downloaded at: https://www.mdpi.com/article/10.3390/molecules27154676/s1. Table S1: List of cancer-associated A2AAR mutations investigated in this study, Figure S1: Homologous displacement of three concentrations of [3H]ZM241385 conducted by increasing the concentrations of ZM241385 in mutant A2AARs, Figure S2: Representative graph of vehicle-normalized cell indices after stimulation with different concentrations of NECA of the mutant A_2A_ARs in a label-free impedance-based cell-morphology assay.
