# Supplementary material for: Cancer-Associated Mutations of the Adenosine A2A Receptor Have Diverse Influences on Ligand Binding and Receptor Functions
Source: Molecules. 2022 Jul 22;27(15):4676. doi: 10.3390/molecules27154676 (PMC9331671; doi:10.3390/molecules27154676)
Supplement: Supplementary file 1 [file molecules-27-04676-s001.zip › molecules-1726179-SI.pdf]

Table S1. List of cancer-associated $A_{2A}AR$ mutations investigated in this study.

Mutation	Affected cases per cancer type across the GDC	Cancer type
A15S$^{1.41}$	1/987	Breast invasive carcinoma
F70L^{ECL1}	1/533	Uterine Corpus Endometrial Carcinoma
I92M$^{3.40}$	1/1060	Lung adenocarcinoma
L95F$^{3.43}$	1/1060	Lung squamous cell carcinoma
S132L$^{4.53}$	3/533<br>1/399	Uterine Corpus Endometrial Carcinoma (3 cases)<br>Colon adenocarcinoma (1 case)
A165V^{ECL2}	1/399	Colon adenocarcinoma
I251T$^{6.53}$	1/399	Colon adenocarcinoma
F257L$^{6.59}$	1/533	Uterine Corpus Endometrial Carcinoma
A265S^{ECL3}	1/987	Breast invasive carcinoma
A265V^{ECL3}	1/987	Breast invasive carcinoma
*A265T^{ECL3}	1/1060	Lung squamous cell carcinoma
V275A$^{7.40}$	1/533	Uterine Corpus Endometrial Carcinoma
H278N$^{7.43}$	1/399	Colon adenocarcinoma
*S281L$^{7.46}$	1/533<br>1/79	Uterine Corpus Endometrial Carcinoma (1 case)<br>Rectum adenocarcinoma (1 case)
P285L$^{7.50}$	1/1060	Lung squamous cell carcinoma

Mutations are shown in the numbering of $A_{2A}AR$ amino acid sequence as well as the Ballesteros and Weinstein number in superscript.

* $A_{2A}AR$ Mutations that were also identified as natural variants.

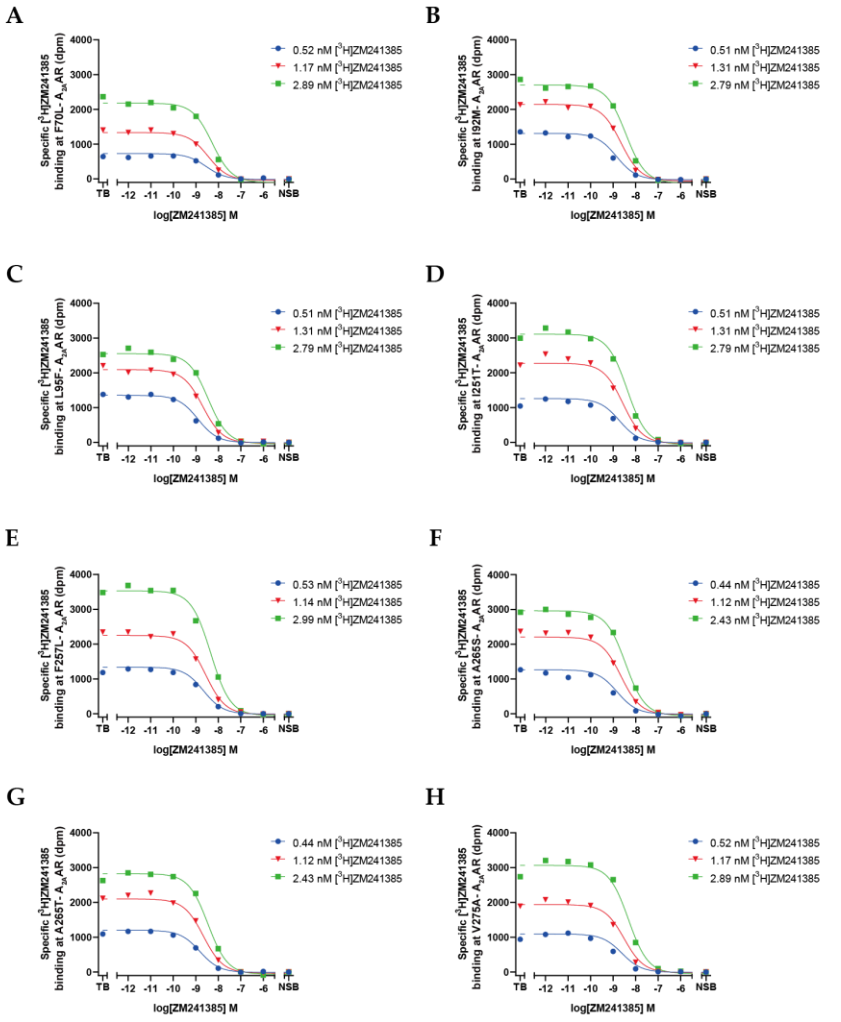

Figure S1. Homologous displacement of three concentrations of $[^3H]$ZM241385 by increasing concentrations of ZM241385 at mutant A_{2A}ARs. Different concentrations of membranes were used dependent on receptor expression levels: (A) F70L-A_{2A}AR (4.5 µg); (B) I92M-A_{2A}AR (4 µg); (C) L95F-A_{2A}AR (3 µg); (D) I251T-A_{2A}AR (3 µg); (E) F257L-A_{2A}AR (1.5 µg); (F) A265S-A_{2A}AR (1.5 µg); (G) A265T-A_{2A}AR (1 µg); (H) V275A-A_{2A}AR (2 µg). Representative curves from one experiment performed in duplicate.

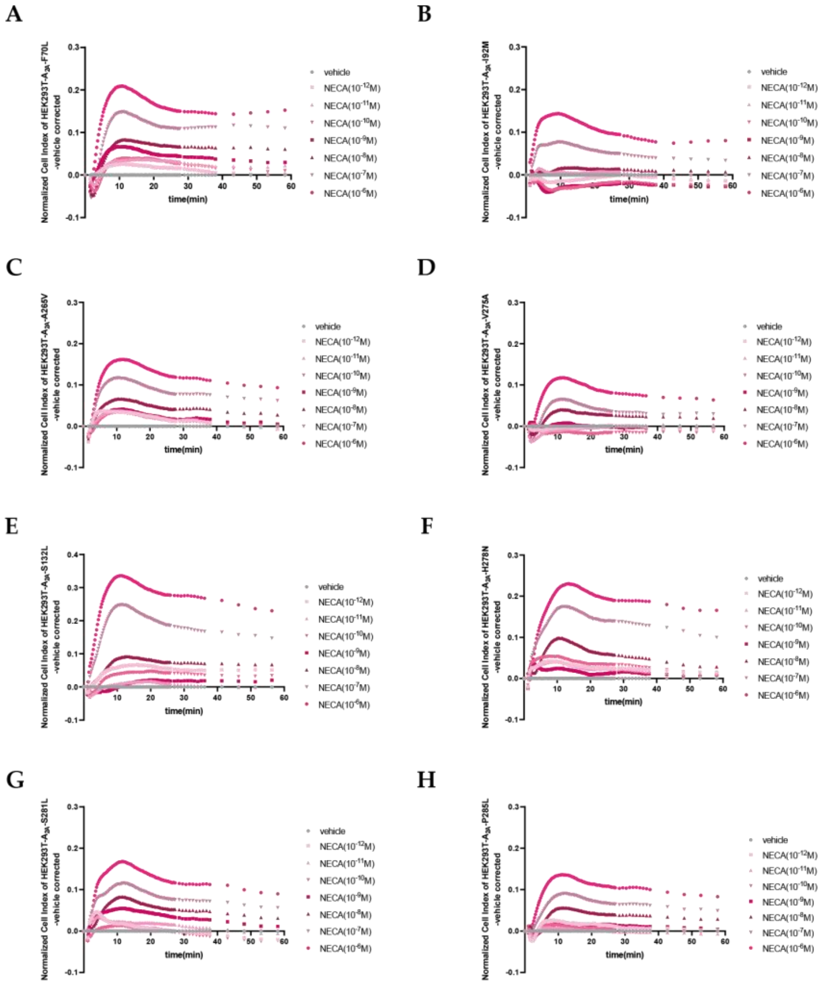

Figure S2. Representative graph of vehicle-normalized cell index after stimulation with different concentrations of NECA at the mutant A_{2A}ARs in a label-free impedance based cell morphology assay. (A) F70L-A_{2A}AR; (B) I92M-A_{2A}AR; (C) A265V-A_{2A}AR; (D) V275A-A_{2A}AR; (E) S132L-A_{2A}AR; (F) H278N-A_{2A}AR; (G) S281L-A_{2A}AR; (H) P285L-A_{2A}AR.
